# Targeting of Liposomes via PSGL1 for Enhanced Tumor Accumulation

**DOI:** 10.1007/s11095-012-0875-5

**Published:** 2012-09-20

**Authors:** Robert Carlisle, Leonard W. Seymour, Constantin C. Coussios

**Affiliations:** University of Oxford, Oxford, UK

**Keywords:** drug delivery, liposome, PSGL1, selectin, tumor

## Abstract

**Purpose:**

To improve the delivery of liposomes to tumors using P-selectin glycoprotein ligand 1 (PSGL1) mediated binding to selectin molecules, which are upregulated on tumorassociated endothelium.

**Methods:**

PSGL1 was orientated and presented on the surface of liposomes to achieve optimal selectin binding using a novel streptavidin-protein G linker molecule. Loading of PSGL1 liposomes with luciferin allowed their binding to e-selectin and activated HUVEC to be quantified *in vitro* and their stability, pharmacokinetics and tumor accumulation to be tested *in vivo* using murine models.

**Results:**

PSGL1 liposomes showed 5-fold (*p* < 0.05) greater selectin binding than identically formulated control liposomes modified with ligand that did not contain the selectin binding domain. When added to HUVEC, PSGL1 liposomes showed >7-fold (*p* < 0.001) greater attachment than control liposomes. In *in vivo* studies PSGL1 liposomes showed similar stability and circulation to control liposomes but demonstrated a >3-fold enhancement in the level of delivery to tumors (*p* < 0.05).

**Conclusions:**

The technologies and strategies described here may contribute to clinical improvements in the selectivity and efficacy of liposomal drug delivery agents.

**Electronic supplementary material:**

The online version of this article (doi:10.1007/s11095-012-0875-5) contains supplementary material, which is available to authorized users.

## INTRODUCTION

Actively targeting drug loaded liposomes to surface receptors up-regulated on tumor cells should improve anti-tumor efficacy. However, despite the assistance provided by the enhanced permeability retention effect (1) there are still many limitations to achieving such targeting. The high interstitial pressure and dense extracellular matrix within tumors (2), makes penetration beyond the perivascular region inefficient (3–5), thereby lowering the opportunity for interaction between targeted liposomes and target cells. Furthermore the receptors available as targets often lack the desired specificity or presentation on tumor cells and show variable expression between different cancer types.

In response to these limitations, targeting therapeutics to the endothelial cells which support tumors has emerged as an attractive alternative strategy. In contrast to normal endothelium, tumor-associated endothelial cells have an inflamed phenotype due to activation by cytokines produced by the tumor (6). This pro-inflammatory activation promotes increased proliferation and results in up-regulated levels of cell surface receptors such as αvβ3 integrins, vascular endothelial growth factor receptors, vascular cell adhesion proteins and selectins (7–10). The properties and surface receptor expression profiles of tumor-associated endothelium are common amongst patients with the same type of cancer (11) and amongst different types of cancer (12–14), and yet still distinct from normal endothelium. Therefore, targeting these receptors should enable the development of therapeutics which are active against a wide range of cancer types whilst maintaining their selectivity for tumor-associated rather than normal endothelium. It is also noteworthy that intravenously delivered therapeutics have rapid and direct contact with tumor-associated endothelium.

Targeting to selectins expressed on endothelial cells is a particularly attractive strategy. Selectins are a family of calcium dependent type-I transmembrane protein comprised of: platelet (P)-selectin (expressed on endothelial cells as well as platelets), endothelium (E)-selectin and leukocyte (L)-selectin. P and E-selectin play a crucial role in recruitment of leukocytes to inflamed endothelium, a process which takes place despite the presence of shear flow and multiple other bloodstream components. This is possible because selectin mediated binding triggers a mechanism of ‘rolling’ and slowing of leukocytes (15,16). Selectins may therefore be ideal target receptors as their physiological role so closely matches the requirements needed to achieve efficient binding of blood borne therapeutic agents to the tumor-associated endothelium. Indeed, success in achieving selectin mediated endothelial targeting in pre-clinical models has been previously reported for viral and non-viral gene delivery vectors (15,16). It is notable that in these reports care was taken to ensure that correct ligand presentation and orientation were achieved to permit optimal receptor binding. The application of protein A or G has allowed antibodies or ligands bearing the Fc domain of immunoglobulin to be linked to therapeutics with high affinity whilst preserving receptor binding regions (17,18). Similar strategies have also been reported utilising the even higher affinity interaction between bungarotoxin and its ligand (19).

Here we report the first *in vivo* use of P-selectin glycoprotein ligand 1 (PSGL1) to enhance the targeting of liposomes to tumor endothelium. We demonstrate that by designing a novel conjugation strategy which presents PSGL1 on the surface of the liposome in the correct orientation specific binding to endothelial cells *in vitro* and enhancements in tumor accumulation *in vivo* can be achieved.

## MATERIALS AND METHODS

### Materials

Hydrogenated soy phosphatidylcholine (HSPC, 840058) and 1,2-distearoyl-*sn*-glycero-3-phosphoethanolamine-N-[biotinyl(polyethyleneglycol)-2000] (DSPE-PEG-Biotin, 880129) were purchased from Avanti Polar Lipids (USA). Cholesterol (C8667), Streptavidin (S4762) and protein G (19459) were purchased from Sigma (UK). Biotinylated-horse radish peroxidise (43-2040) was purchased from Invitrogen (CA, USA). Goat IgG-horse radish peroxidise (12348) was purchased from Millipore (UK). Fc linked PSGL1 (3345-PS) and the control Fc lacking the PSGL1 domain (110-HG) were purchased from R and D Systems (UK). Human Umbilical Vein Endothelial cells (HUVECs; PromoCell, Germany) were maintained in EGMTM-2-Endothelial Cell Medium-2 (Cambrex Bio Science, USA), supplemented with hydrocortisone, h-FGF-B, VEGF, R3-IGF-1, ascorbic acid, heparin, FBS, hEGF, and GA-1000 (Cambrex Bio Science, USA). E-selectin was purchased from R and D systems. E1, E3-deleted Ad5 expressing cytomegalovirus immediate-early (CMVIE) promoter-driven luciferase (Adluc) was purchased from Hybrid Biosystems Ltd (UK). D-Luciferin and B16-F10-luc cells were purchased from Caliper life sciences (USA) and maintained as recommended by the manufacturer.

### Formulation of Liposomes and Luciferin Loading

Liposomes were formulated by mixing HSPC, cholesterol and DSPE-PEG or DSPE-PEG-biotin at ratios of 55:40:5 in 2 chloroform: 1 methanol, in a round bottomed flask. After complete dissolution rotary evaporation for 30 min at 55°C achieved a thin film which after drying under high vacuum overnight was rehydrated in 300 mM Tris pH 10 for 60 min. Sizing was achieved by extrusion at 55°C using a LiposoFast extruder (purchased from GC technology Ltd, UK) with 11 passes through a 400 nm filter (86-039) followed by a 11 passes through a 100 nm (86-037) filter. Buffer exchange into 25 mM citrate, 150 mM NaCl buffer pH 5 using a PD10 column (GE healthcare) created a trans-bilayer pH gradient which was used to actively load luciferin dissolved in 25 mM citrate, 150 mM NaCl buffer. After incubation of 1.8 mL of 20 mg/mL liposomes with luciferin (30 mg in 100 μL) overnight at room temperature, non-loaded luciferin was removed and buffer exchanged for phosphate buffered saline. Luciferin loading was calculated by the addition of a 1:1 mix of buffer containing free recombinant luciferase (25 mM HEPES, 2 mM EDTA, 60 μg/mL BSA, 40 μg/mL DTT, 20 μg/mL luciferase (Sigma, F3776), pH 7.5) and an ATP buffer (25 mM HEPES, 2 mM EDTA, 1 mM ATP, pH 7.5). Measurements were taken in a luminometer (Berthold, LB9507) following heating sample to 100°C for 5 min or not and compared to a standard curve of identically treated dilutions of free luciferin. The percentage of encapsulated luciferin was calculated by the formula ((μg post heating – μg pre-heating) / μg post heating) × 100. After purification and buffer exchange into PBS using a PD column the percentage of encapsulated luciferin was found to be in excess of 97%. Dynamic light scattering was performed using a Malvern Zetasizer 3000 (Malvern Instruments), with 3 sub-runs of 10 and automatic analysis, on 3 separate samples.

### Formulation and Testing of a Protein G-Streptavidin Linker and Addition to Liposomes

Streptavidin was modified with protein G at a range of ratios using EDC chemistry as modified from (20). Streptavidin 0.1 mg was dissolved in 100 μL (final conc 0.013 mM) of 0.1 M MES (2-[morpholino]ethanesulfonic acid), 0.1 M NaCl, buffer pH 5.6 and 10 μL 0.04 mg (2.5 mM final conc), of 1-ethyl-3-(3-dimethylaminopropyl)-carbodiimide hydrocholoride (EDC, Pierce) and Sulfo-NHS (1 mg, 5 mM final conc) was then added and after 15 min at room temperature buffer pH was raised using PBS. Protein G was then added at a range of ratios for 2 h at room temperature, as defined in figure legends. Analysis of changes to protein size and ligand binding ability was performed using SDSPAGE with Coomassie staining or transfer onto nitrocellulose and blotting with biotinylated-horse radish peroxidise (HRP) (Invitrogen) or Fc-HRP (Millipore) in 5% milk/PBS for 1 h, followed by 3 × 10 min washes in PBS/Tween 0.01%. Nitrocellulose was developed with ECL reagent (GE Healthcare) and images captured and densiometrically analysed using an Alpha Innotech imager and FluorChemHD2 software. The ratio which gave optimal maintenance of binding to both Fc and biotin was identified (1 streptavidin : 2 protein G) and used for addition to luciferin loaded liposomes incorporating PEG-biotin (see Formulation of Liposomes and Luciferin Loading). After incubation overnight excess protein G-streptavidin was removed by separation on a PD10 column (GE healthcare).

### *In Vitro* Assays of Binding to Selectins

Maxisorb ELISA plates (Nunc) were coated with E-selectin (R and D systems) as described by (21). Optimisation and confirmation of coating was provided using a BCA assay (Sigma). E-selectin (50 μg/mL), dissolved in PBS was added 50 μL/well overnight at 4°C, then removed before blocking with 100 μL PBS/1% bovine serum albumin for 1 h at room temperature. After three washes in 200 μL of PBS, 50 μL of luciferin loaded Fc-control or PSGL1 liposomes in PBS was added per well for 30 min at room temperature. Sample was then removed and 10 washes in 200 μL of PBS performed. 200 μL of PBS was placed in each well and the plate heated to 70°C degrees. Sample was transferred into luminometer tubes, luciferase/ATP was added as in Formulation of Liposomes section and measurement performed using a luminometer (Berthold, LB9507). For assaying binding to HUVEC, cells were plated at 10,000 per well in a black 96 well plate. To achieve luciferase expression in the HUVECs so that the binding of luciferin loaded liposome could be assayed the cells were infected with an adenovirus vector expressing the luciferase transgene (Adluc). After 24 h, 500 of particles of Adluc were added per cell for 90 min before washing and returning to the incubator overnight. Liposomes (2 μL per well) were then added in 100 μL fresh media. The luciferase/luciferin signal was then assayed at 5, 10, 20 and 60 min using a Wallac 1420 plate reader. 5 × washes in 100 μL PBS were then performed and media was replaced and incubation and measurement continued for the next 27 h. Experiments were performed with *n* = 4, results were typical of three separate experiments and analysis of significance was obtained using ANOVA comparisons and PRISM software.

### *In Vivo* Analysis of Release, Circulation, and Tumor Accumulation

B16-F10-luciferase cells were grown and implanted (5 × 10^5^ cells) into the flank of C57 Bl6 mice. After 3 weeks when tumors had reached a size of between 500 and 700 mm^3^ their luciferase expression was confirmed and compared by injecting 100 μL of 15.8 mg/mL of free luciferin in PBS intravenously and imaging after 5 min using an IVIS 100 (Caliper Life Sciences, USA). 2 days later mice were re-imaged to verify their return to background luminescence levels and then 80 μL liposomes / 280 μg of luciferin sample was injected intravenously. IVIS images and blood samples (20 μL) were taken at 15, 30, 60 and 120 min after injection after which time mice were culled and organs harvested for quantification of biodistribution and tumor accumulation. Samples were lysed and heated and luciferin content calculated against standard curves made with identically processed organs from untreated mice which had been spiked with serial dilutions of known amounts of luciferin loaded liposomes. Heating was demonstrated to both release still encapsulated luciferin and destroy endogenous tumor luciferase. Organ and blood associated luciferin could therefore be calculated by the addition of luciferase/ATP as in Formulation of Liposomes section. Data shown were typical of two experiments. Analysis was performed by ANOVA using PRISM.

## RESULTS

### Protein G-Streptavidin Conjugates with Ligand Binding Properties of Both Sub-Units

**Fig. 1:**
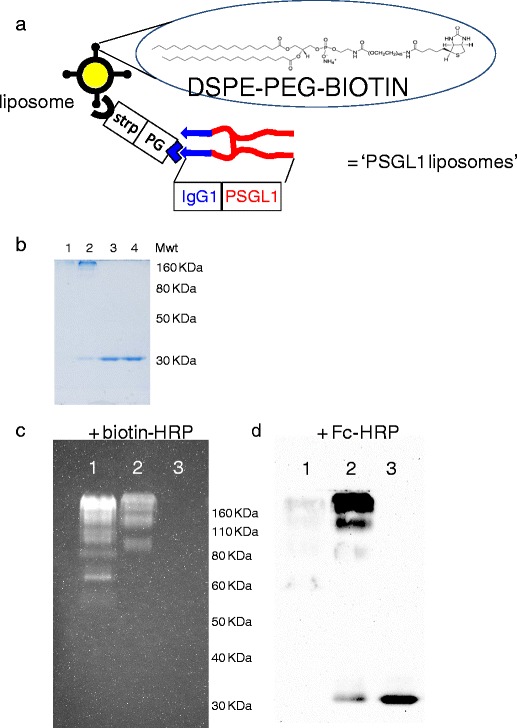
Production of a protein G-streptavidin conjugate for linkage of immunoglobulin G Fc containing ligands to liposomes. (**a**) Outline of the strategy for the linkage of liposomes presenting PEG-biotin to the targeting ligand PSGL1-Fc via a linker comprised of streptavidin (for biotin binding) and protein G (for Fc binding). (**b**) Coomassie stained SDSPAGE of protein G-streptavidin conjugates formed at ratios of; lane 1 = 3 streptavidin : 1 protein G, lane 2 = 1 streptavidin : 4 protein G, lane 3 = free protein G in reaction buffers, lane 4 = free protein G direct from stock. (**c**) Probing of nitrocellulose with biotin-HRP; lane 1 = 3 streptavidin : 1 protein G, lane 2 = 1 streptavidin : 4 protein G, lane 3 = free protein G in reaction buffers. (**d**) Probing of nitrocellulose with Fc-HRP; lane 1 = 3 streptavidin : 1 protein G, lane 2 = 1 streptavidin : 4 protein G, lane 3 = free protein G in reaction buffers.

The commercial availability of DSPE-PEG-biotin makes the streptavidin-biotin interaction an attractive and viable proposition when considering methods of achieving attachment of retargeting ligands to liposomes. Similarly the commercial availability of a P-selectin glycoprotein ligand-1 (PSGL1) fusion protein bearing human IgG Fc regions provides the possibility of linking this retargeting agent via its interaction with protein A or G, as reported for non-viral and viral gene delivery agents (17,22). However mechanisms of linking streptavidin binding to protein G binding are not presently available and so we developed the strategy outlined (Fig. 1a), and in so doing created a novel biomaterial with this dual binding functionality. The use of EDC/SufoNHS chemistry allowed the rapid and reproducible production and testing of functional conjugates (Fig. 1b–d). The complete absence of a band of unmodified protein G (at approximately 30 kDa) in lane 1 of Fig. 1b suggests that at this ratio (3 streptavidin : 1 protein G) 100% of the protein G has been bound to streptavidin. Whilst the faint band at 30 kDa present in lane 2 indicates that at a ratio of 1 streptavidin : 4 protein G, not all the protein G was conjugated. Indeed, further analysis (Fig. S1a) showed that at this ratio 24% of the protein G remained unmodified. The presence of high molecular weight bands in Fig. 1b, lanes 1 and 2 (>160 kDa) confirmed the formation of protein G-streptavidin conjugates. In control lanes 3 and 4, which contained sample with no streptavidin, the protein G migrated to its predicted weight. Blotting onto nitrocellulose enabled the capacity of the conjugates to bind to biotin (Fig. 1c) or Fc domains (Fig. 1d) to be assayed. At a ratio of 3 streptavidin to 1 protein G (lane 1) intense binding was evident at several molecular weights between 80 and 200 kDa (Fig. 1c). A band of unmodified streptavidin was also detected at about 70 kDa. The lower intensity of these bands in lane 2 indicated that the increased level of protein G in these conjugates had diminished the ability of the streptavidin to bind to biotin. Notably no staining was evident in lanes 3 demonstrating no cross-reactivity of protein G with biotin-HRP (Fig. 1c). The conjugates in lane 1 showed decreased binding compared to those in lane 2 when probed with Fc-HRP (Fig. 1d). As a result of these studies the ratio of 1 streptavidin : 2 protein G was identified as the optimal to maintain high binding capacity to both biotin and Fc (Fig. S1b). Although this ratio still resulted in a small level (6%) of protein G remaining unmodified, this was removed by size exclusion chromatography before addition of PSGL1 to liposomes-streptavidin-protein G. These data confirmed the creation of a novel biomaterial which could be used in combination with biotinylated liposomes to create a platform vector for the testing of any targeting strategy using any Fc bearing molecule (e.g. antibodies, fusion proteins).

### PSGL1 Modification of Liposomes for Selectin and HUVEC Binding

**Fig. 2:**
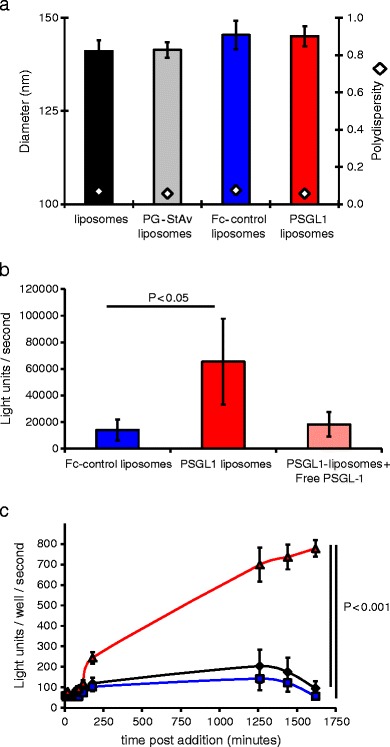
The influence of PSGL1 addition on the physicochemical and biological activity of liposomes. (**a**) Dynamic light scattering analysis of the size and polydispersity of liposomes or modified liposomes. (**b**) Ability of Fc-control or PSGL1 liposomes to adhere to E-selectin coated plates, as measured by detection of luminescence in wells after incubation with luciferin loaded liposomes, washing, rupture of liposomes and luciferase enzyme addition. *N* = 3, SD shown, significance (*p* < 0.05) calculated by ANOVA. (**c**) Ability of liposomes to adhere to / deliver luciferin to HUVECs expressing the luciferase enzyme, as assayed after incubation with luciferin loaded liposomes, washing, and multiple subsequent luminescence measurements. Black line and diamonds = unmodified liposomes, blue line and squares = Fc control liposomes, red line and triangles = PSGL1 liposomes. *N* = 4, standard error (se) shown, significance (*p* < 0.001) calculated by ANOVA with Tukey post test all samples comparison using PRISM.

Dynamic light scattering was used to assess the influence of ligand addition on the stability, size and monodispersity of liposomes. Throughout the stages of modification with protein G-streptavidin, purification, and then addition of PSGL1 or ‘Fc-control’ protein, liposomes maintained a polydispersity below 0.1 and a size of approximately 150 nm (Fig. 2a). This size is desirable for intravenously delivered agents as it is above the cut-off of the hepatic endothelial fenestrae in pre-clinical models and patients (23,24). This should provide liposomes with reduced hepatic clearance, extended circulation and therefore enhanced interaction with tumor associated endothelium. Repeat DLS analysis of liposomes after one month storage showed maintenance of these size and polydispersity values, demonstrating that surface modification with PSGL1 or control ligand had not detrimentally influenced stability.

The interaction between the enzyme luciferase and its substrate luciferin produces dose dependent quantifiable photon emissions. Loading of liposomes with luciferin therefore enabled their binding to selectin coated plates to be assayed with great sensitivity (see Materials and Methods). Following a 1 h incubation with liposomes and thorough washing with PBS, binding was quantified by heating the plate to release luciferin which was then added to recombinant luciferase enzyme and assayed by luminometry. PSGL1 liposomes showed 5-fold (*p* < 0.05) greater binding to the selectin coated plates than control Fc-liposomes (Fig. 2b). This binding could be inhibited by pre-incubation of the plate with a 100-fold excess of free PSGL1.

The specific PSGL1 mediated targeting of liposomes was confirmed in studies using HUVEC expressing luciferase (Fig. 2c). After a period of 60 min to allow interaction between cells and liposomes, cells were thoroughly washed to remove any non-bound liposomes. During the subsequent 60 min no difference in luciferin luminescence, was observed for cells exposed to PSGL1 liposomes compared to those exposed to Fc control liposomes or non-modified liposomes, perhaps indicative of the need for a period to allow release of luciferin from PSGL1 liposomes bound to cells. However, from 3 h onwards cells exposed to PSGL1 liposomes started to produce significantly increased luminescence signal compared to controls, indicative of improved binding. This differential increased over time reaching 7-fold by 27 h (*p* < 0.001). In previous reports of *in vitro* selectin mediated targeting, binding required activation of the endothelial cells with tumour necrosis factorα (TNFα) (25). Notably the targeting observed in our studies was achieved without the addition of TNFα. This suggests that, in accordance with previous reports (26), the use of Adluc to achieve luciferase expression in these cells was a sufficient stimulus to also induce selectin presentation. In studies where delivery was compared with and without a 4 h pre-incubation with 100 ng/mL of TNFα, similar results to those in Fig. 2c were obtained (Fig. S2b). Exposure of HUVEC to Adluc or TNFα and probing with Fc-control or PSGL1 confirmed the presence and ligand binding functionality of selectins under these conditions (Fig. S2c).

### *In Vivo* Testing of PSGL1 Liposomes

**Fig. 3:**
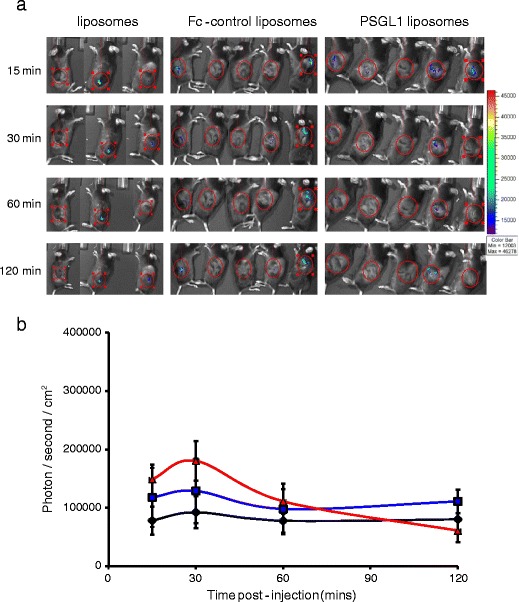
Stability of liposomes following intravenous delivery to mice bearing B16-F10-luciferase tumors. (**a**) An IVIS system was used to capture luminescence from luciferase tumors at 15, 30, 60 and 120 min post injection of 80 μL/280 μg of luciferin loaded liposomes. (**b**) Luminescence signal from tumors was quantified and plotted against time for the 3 liposome formulations. Black line and diamonds = unmodified liposomes (*n* = 3), blue line and squares = Fc control liposomes (*n* = 5), red line and triangles = PSG1 liposomes (*n* = 4), se shown, no significant differences were evident as calculated by ANOVA.

**Fig. 4:**
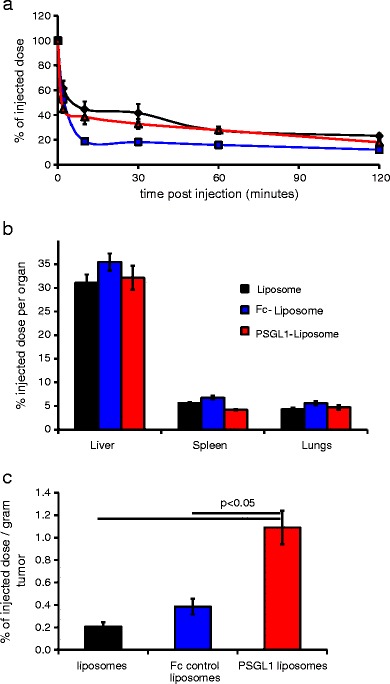
Pharmacokinetics and organ and tumor accumulation of liposomes following intravenous delivery to mice bearing B16-F10-luciferase tumors. (**a**) Blood samples collected at 5, 10, 30, 60, 90 and 120 minutes were analysed for luciferin content by heating followed by luciferase enzyme addition and luminometry. Black line and diamonds = unmodified liposomes (*n* = 3), blue line and squares = Fc control liposomes (*n* = 5), red line and triangles = PSG1 liposomes (*n* = 4), se shown, no significant differences at 120 min were evident as calculated by ANOVA. (**b**) Liposome accumulation in specified organs was quantified at 120 min post injection by homogenising organs heating, adding luciferase enzyme and measuring by luminometry. Unmodified liposomes *n* = 3, Fc control and PSGL1 liposomes *n* = 5, data calculated as % of injected dose / whole organ. (**c**) Liposome accumulation in tumors was quantified at 120 min post injection by homogenising tumors, heating, adding luciferase enzyme and measuring by luminometry. Unmodified liposomes *n* = 3, Fc control and PSGL1 liposomes *n* = 5, data calculated as % of injected dose / gram of tumor (%ID/g), significant difference (*p* < 0.05) evident as calculated by ANOVA with Tukey post test all samples comparison using PRISM.

In addition to enabling simple, accurate and sensitive measurement of activity *in vitro* as described above, loading of liposomes with luciferin also enabled *in vivo* activity to be characterised. C57BL6 mice bearing B16-F10-luciferase tumors were a good model for these studies as they allowed pre-testing and validation with non-encapsulated free luciferin to ensure that no significant inter or intra-group differences in tumor size or vascular supply existed (Fig. S3a and S3b). After 48 h to allow the free luciferin to clear, these mice were then dosed intravenously with unmodified liposomes, Fc control liposomes or PSGL1 liposomes. For 2 h post injection images were taken and analysed using an IVIS system to characterise liposome stability and blood samples were taken to provide pharmacokinetic data. It is clear that within the 2 h time-frame of the experiment no significant inter or intra-group differences in the signal from the B16-F10-luciferase tumors were evident (Fig. 3 a and b). This demonstrates that over this duration the luciferin within the non-modified liposomes, Fc-control liposomes and PSGL1 liposomes remained similarly encapsulated, indicating that all the formulations possessed similar stability in the bloodstream. In accordance with these findings, in studies using liposomes with a similar composition to the non-modified liposomes used here, Kheirolomoom *et al.* calculated a luciferin release rate which approached zero after 30 min (27). The 2 h time-frame of the experiments described here was chosen to allow specific PSGL1 mediated binding to tumor associated endothelia to be distinguished from EPR mediated passive accumulation in the tumor, the effects of which are usually assayed at 24 or 48 h (28). Pharmacokinetic studies revealed that non-modified liposomes and PSGL1 liposomes showed almost identical blood clearance profiles with bloodstream concentrations which were halved 10 minutes after injection and then stabilised to 20% of the injected dose over the next 110 min (Fig. 4a). This profile suggests a saturable first pass hepatic clearance mechanism, perhaps mediated by kupffer cell capture. Notably the clearance of Fc-control liposomes followed the same profile but a concentration of just 10% of the injected dose was recovered from the bloodstream at 2 h. In studies using liposomes with a similar composition to the non-modified liposomes used here, Kheirolomoom *et al.* reported the same biphasic clearance profile (27). Analysis of liver, spleen and lung accumulation supported a role for first pass hepatic clearance with over 30% of the injected dose being recovered from the liver in all treated mice (Fig. 4b). Liver accumulation occurred despite the inclusion of polyethylene glycol (PEG) in the liposomes tested here. Whilst such PEGylation did provide much reduced liver capture compared to non-PEGylated liposomes (data not shown), reducing this capture further by increasing the percentage of PEGylation used may allow further enhancements in circulation and tumor accumulation.

Post-cull analysis of tumor accumulation demonstrated that approximately 1.2% of the injected dose of PSGL1 liposomes had accumulated per gram of tumor (1.2% ID/g), during the 2 h following intravenous administration. This proved to be a significant (*p* < 0.05) and substantial (5-fold or 3.5-fold) increase compared non-modified liposomes or Fc-control liposomes (Fig. 4c). As all these liposome formulations demonstrated identical bloodstream stability and similar clearance profiles this increase is indicative of PSGL1 ligand providing increased association with tumor-associated endothelium. Probing of tumor cryosections with PSGL1 confirmed the presence of selectins on this endothelium (Fig. S3c). The level of tumor association of non-modified liposomes is in accordance with the levels reported in pre-clinical studies which have utilised radiolabelling to demonstrate that Doxil like formulations achieve 0.6% ID/g tumor after 18 h (29) or 0.2% ID/g tumor after 4 h (30). Recently a strategy for targeting liposomes directly to tumors achieved an impressive 4.7% ID/g of tumor after 48 h (31), an improvement over the level observed in our studies. This improvement may be due to the longer duration allowed before assaying and the enhanced ability to achieve efficient internalisation. The different characteristics of the target tumor models used may also be an important factor, as recently demonstrated by Ogawara *et al.* (32). The protein G-streptavidin ‘platform’ vector and *in vivo* test system we have developed could allow a vast range of Fc containing antibodies or fusion proteins to be directly compared, so that optimal targeting strategies, conditions and ligands could be identified. Although the high immunogenicity of streptavidin may limit its ultimate clinical application the optimal targeting ligands identified by these studies could be formulated as direct conjugates to the liposomes.

## DISCUSSION

The advantages of targeting drug delivery to tumor-associated vasculature has long been recognised (33). Selectin molecules provide a particularly attractive target due to their upregulation on inflamed endothelium and the fact that their physiological role in leukocyte recruitment mimics that required to bind and slow drug delivery macromolecules under conditions of shear flow. As a result, several investigations using a variety of selectin ligands to target liposomes have been reported (34) (25) (35), with impressive *in vitro* results. Here we present the first demonstration that such targeting can improve tumor accumulation in *in vivo* studies performed using pre-clinical tumor bearing models. The reported difficulties of synthesising and using sialyl Lewis(X) (36) and our previous experience comparing anti-selectin antibodies to the PSGL1 ligand for retargeting of adenovirus vectors (17) persuaded us that the PSGL1 ligand would provide optimal ease of use, efficiency of targeting and reduced immunogenicity. Previous studies have also demonstrated the advantage of attaching targeting ligand to therapeutic agent in ways that use epitopes of the ligand that are not involved in receptor binding and achieve appropriate orientation of the ligand (37) (19). To meet this requirement we developed a new protein G-streptavidin biomaterial as a linker between biotin on the liposome surface and Fc regions in the PSGL1. This conjugate was produced by chemically cross-linking the two recombinant proteins. We believe the chemical conjugation strategy we employed to be the most efficient and assured method considered the challenges faced in trying to engineer and express recombinant streptavidin fusion proteins. Although the problems of inclusion body formation and inappropriate refolding of native streptavidin produced in *E. coli* have been overcome (38,39), in the case of fusion proteins the yield of correctly folded protein is often sub-optimal and a general refolding method has not been established. A sophisticated enzyme mediated approach to circumvent this problem and produce a GFP-streptavidin fusion protein has recently been published, but this has not yet been attempted for a protein G-streptavidin (40). Our optimised protein G-streptavidin linker demonstrated binding to both Fc domains and biotin (Fig. 1) and produced a ‘platform’ system that could be used to test the ability of any Fc bearing antibody or fusion protein to target liposomes. In our *in vitro* experiments substantial and significant increases in the attachment of liposomes to E-selectin and activated HUVEC was achieved using PSGL1 (Fig. 2). To date, there have been no reports testing the ability of selectin targeted liposomes to accumulate in tumor-associated endothelium *in vivo*, perhaps because such experiments fell outside the scope of previous studies. In our studies loading liposomes with luciferin allowed *in vivo* stability, pharmacokinetics and tumor accumulation to be sensitively and accurately measured (Fig 3 and 4). Liposomes targeted with PSGL1 showed similar stability and pharmacokinetics to non-targeted controls but demonstrated significantly and substantially increased accumulation in tumors. Levels obtained after just 2 h approached 1.2% of the injected dose per gram of tumor. PEGylated liposomes demonstrate good circulation and EPR assisted tumor accumulation, however their enhanced stability means that they achieve sub-optimal release of therapeutic cargo and poor penetration beyond the perivascular space (3). The application of ultrasound offers a method by which the targeted release of drug payload from such liposomes can be non-invasively triggered (41). This is an especially attractive strategy when combined with the delivery of microbubbles, which allows powerful ‘cavitation’ events to be generated at diagnostic ultrasound pressure (42). However, following co-delivery of liposomes and microbubbles the EPR effect will allow entry of liposomes (100–200 nm) into tumors whilst excluding microbubbles (~2.5 μm), and such partitioning will prevent the microbubbles impacting on the liposomes. By increasing accumulation of liposomes on the tumor-associated endothelium using PSGL1 targeting, it is hoped we can enhance the efficiency of microbubble/ultrasound-induced tumor-specific release of drug from liposomes. Our further studies will investigate the combination of these strategies for enhanced tumor therapy.

## CONCLUSION

Here we describe the novel application of PSGL1 to liposomes to provide successful targeting to selectins. This was achieved by the chemical formulation of a novel biomaterial, a protein G-streptavidin conjugate, which retained the ligand binding activities of both its sub-units and could therefore act as a linker between liposomes and the PSGL1 molecule. The creation of this linker provides the opportunity to test a range of different ligands containing the immunoglobulin Fc region (antibodies, fusion proteins), using the same ‘platform’ protein G-streptavidin modified liposome formulation. This strategy has the advantage of linking ligands to liposomes via domains that are not involved in their interaction with cell surface receptors. Such optimal orientation and presentation proved beneficial in our studies using PSGL1 which provided substantial enhancements in selectin binding *in vitro*. The subsequent significant increases in tumor accumulation we observed using PSGL1 targeted liposomes in a pre-clinical xenograft model, suggests such a strategy may provide clinical improvements in the selectivity and efficacy of liposomal drug delivery agents.

## Electronic supplementary material

Below is the link to the electronic supplementary material.

Fig. S1 (PPTX 2390 kb)

Fig. S2 (PPTX 150 kb)

Fig. S3 (PPTX 1272 kb)

## ABBREVIATIONS

Fc: immunoglobulin fragment crystallizable region
HRP: horse radish peroxidise
HUVEC: human umbilical cord venous endothelial cells
IV: intravenous
PSGL1: P-selectin glycoprotein ligand 1
