# Supplementary material for: Targeting of Liposomes via PSGL1 for Enhanced Tumor Accumulation
Source: Pharm Res. 2012 Sep 20;30(2):352–61. doi: 10.1007/s11095-012-0875-5 (PMC3553414; doi:10.1007/s11095-012-0875-5)
Supplement: Supplementary file 1 — (PPTX 2390 kb) [file 11095_2012_875_MOESM1_ESM.pptx]

## Slide 1
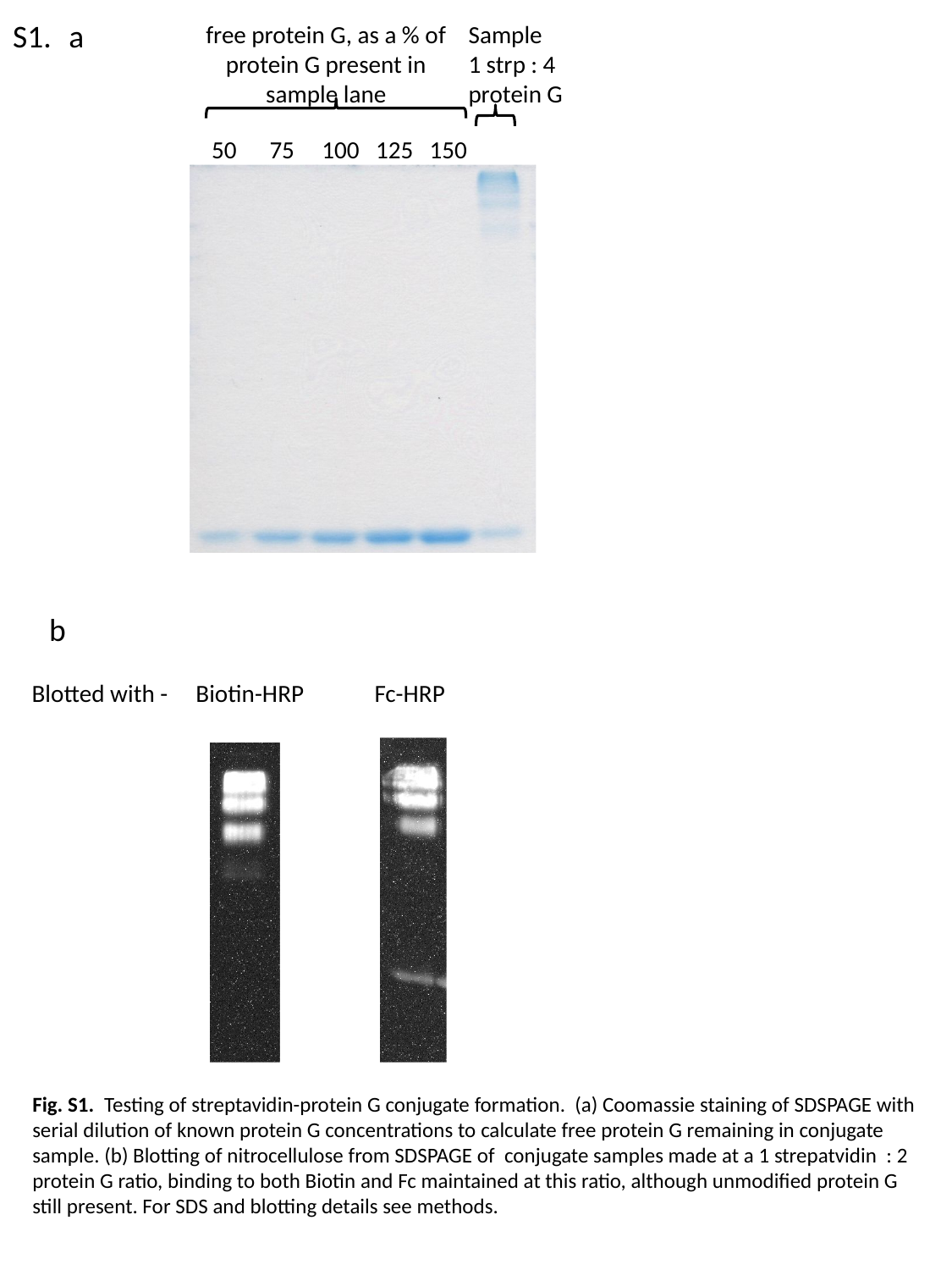

S1.
a
free protein G, as a % of protein G present in sample lane
Sample
1 strp : 4 protein G
 50 75 100 125 150
b
Blotted with -
Biotin-HRP
Fc-HRP
Gel 2 – Fc-HRP
Fig. S1. Testing of streptavidin-protein G conjugate formation. (a) Coomassie staining of SDSPAGE with serial dilution of known protein G concentrations to calculate free protein G remaining in conjugate sample. (b) Blotting of nitrocellulose from SDSPAGE of conjugate samples made at a 1 strepatvidin : 2 protein G ratio, binding to both Biotin and Fc maintained at this ratio, although unmodified protein G still present. For SDS and blotting details see methods.
