# Supplementary material for: Targeting of Liposomes via PSGL1 for Enhanced Tumor Accumulation
Source: Pharm Res. 2012 Sep 20;30(2):352–61. doi: 10.1007/s11095-012-0875-5 (PMC3553414; doi:10.1007/s11095-012-0875-5)
Supplement: Supplementary file 2 — (PPTX 150 kb) [file 11095_2012_875_MOESM2_ESM.pptx]

## Slide 1
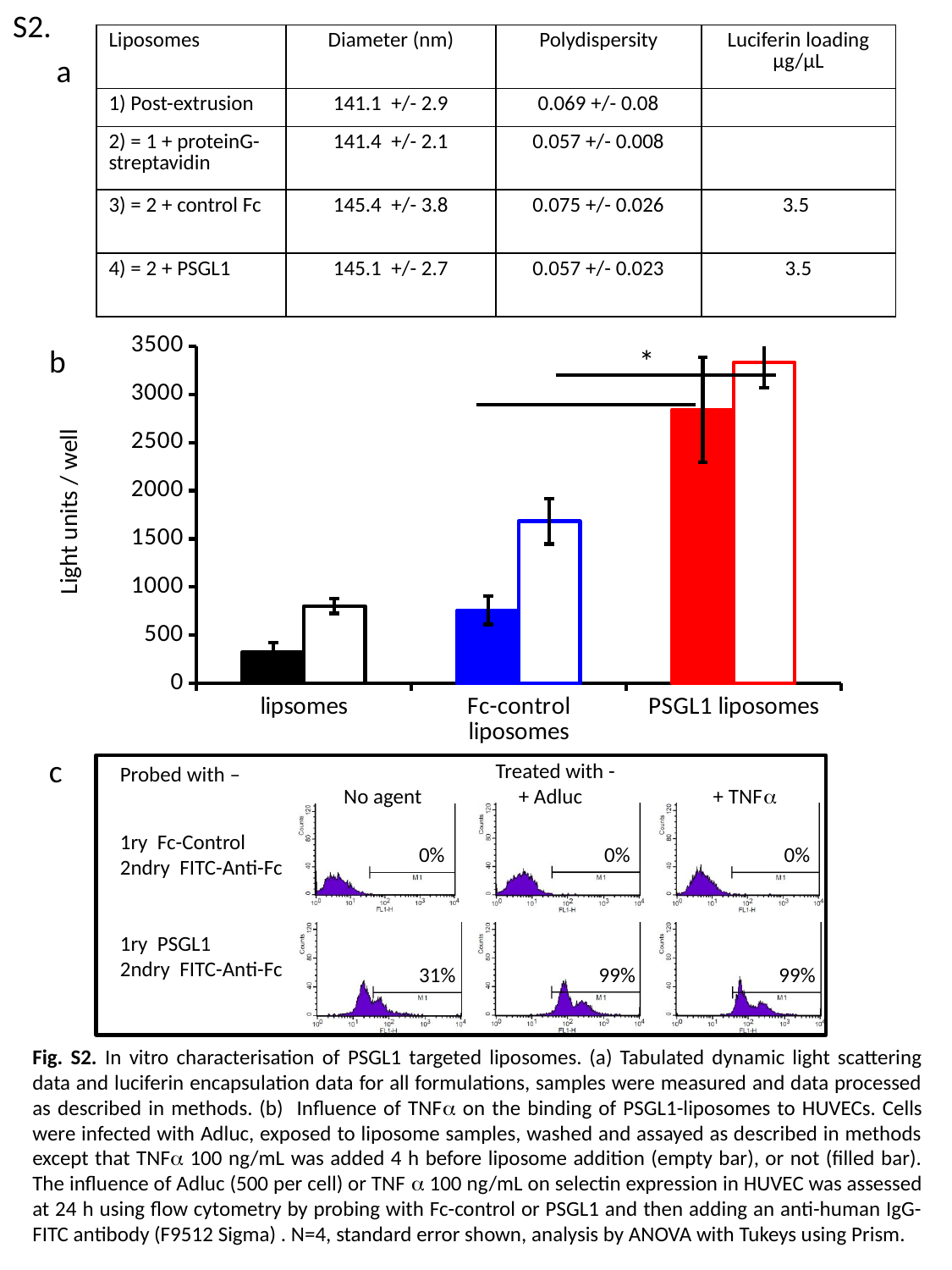

S2.
| Liposomes | Diameter (nm) | Polydispersity | Luciferin loading µg/µL |
| --- | --- | --- | --- |
| 1) Post-extrusion | 141.1 +/- 2.9 | 0.069 +/- 0.08 | |
| 2) = 1 + proteinG-streptavidin | 141.4 +/- 2.1 | 0.057 +/- 0.008 | |
| 3) = 2 + control Fc | 145.4 +/- 3.8 | 0.075 +/- 0.026 | 3.5 |
| 4) = 2 + PSGL1 | 145.1 +/- 2.7 | 0.057 +/- 0.023 | 3.5 |
a
### Chart
| Category | no TNF added | + TNF |
|---|---|---|
| lipsomes | 328.5 | 803.25 |
| Fc-control liposomes | 758.5 | 1683.75 |
| PSGL1 liposomes | 2840.25 | 3331.5 |b
*
Light units / well
c
Treated with -
 No agent + Adluc + TNFα
Probed with –
1ry Fc-Control
2ndry FITC-Anti-Fc
1ry PSGL1
2ndry FITC-Anti-Fc
0%
0%
0%
31%
99%
99%
Fig. S2. In vitro characterisation of PSGL1 targeted liposomes. (a) Tabulated dynamic light scattering data and luciferin encapsulation data for all formulations, samples were measured and data processed as described in methods. (b) Influence of TNFα on the binding of PSGL1-liposomes to HUVECs. Cells were infected with Adluc, exposed to liposome samples, washed and assayed as described in methods except that TNFα 100 ng/mL was added 4 h before liposome addition (empty bar), or not (filled bar). The influence of Adluc (500 per cell) or TNF α 100 ng/mL on selectin expression in HUVEC was assessed at 24 h using flow cytometry by probing with Fc-control or PSGL1 and then adding an anti-human IgG-FITC antibody (F9512 Sigma) . N=4, standard error shown, analysis by ANOVA with Tukeys using Prism.
