# Supplementary material for: Targeting of Liposomes via PSGL1 for Enhanced Tumor Accumulation
Source: Pharm Res. 2012 Sep 20;30(2):352–61. doi: 10.1007/s11095-012-0875-5 (PMC3553414; doi:10.1007/s11095-012-0875-5)
Supplement: Supplementary file 3 — (PPTX 1272 kb) [file 11095_2012_875_MOESM3_ESM.pptx]

## Slide 1
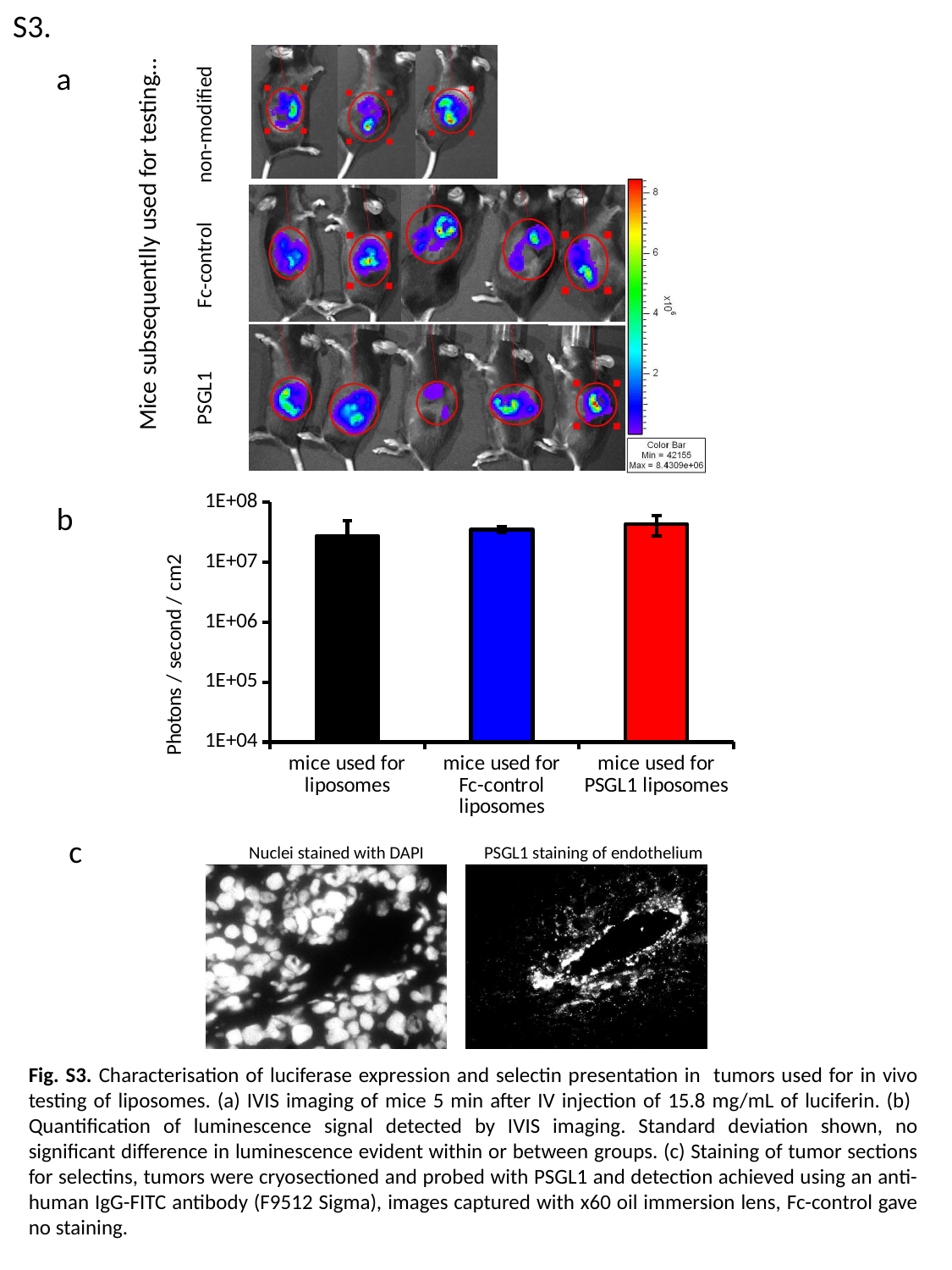

S3.
a
 Mice subsequentlly used for testing…
 PSGL1 Fc-control non-modified
### Chart
| Category | Mean Photons / sec / cm2 |
|---|---|
| mice used for liposomes | 27371333.33333335 |
| mice used for Fc-control liposomes | 35180000.0 |
| mice used for PSGL1 liposomes | 43580000.0 |b
c
Nuclei stained with DAPI PSGL1 staining of endothelium
Fig. S3. Characterisation of luciferase expression and selectin presentation in tumors used for in vivo testing of liposomes. (a) IVIS imaging of mice 5 min after IV injection of 15.8 mg/mL of luciferin. (b) Quantification of luminescence signal detected by IVIS imaging. Standard deviation shown, no significant difference in luminescence evident within or between groups. (c) Staining of tumor sections for selectins, tumors were cryosectioned and probed with PSGL1 and detection achieved using an anti-human IgG-FITC antibody (F9512 Sigma), images captured with x60 oil immersion lens, Fc-control gave no staining.
